# Environmental Sources and Tissue-Specific Selection Jointly Shape the Bacterial Community of the Sea Cucumber (*Apostichopus japonicus*) in Artificial Reef Areas of Laizhou Bay, Bohai Sea

**DOI:** 10.3390/microorganisms14092109

**Published:** 2026-09-20

**Authors:** Shengkai Lin, Qingchen Zhao, Linlin Ma, Tuo Zhou, Hexiang Yang, Zhongxin Wu, Tao Tian, Qingxia Li

**Affiliations:** 1 Center for Marine Ranching Engineering Science Research of Liaoning, Dalian Ocean University, Dalian 116023, China; shengkai3@outlook.com (S.L.); zqc078009@163.com (Q.Z.); 15615504539@163.com (L.M.); 13840467158@163.com (T.Z.); wuzhongxin@dlou.edu.cn (Z.W.); 2 College of Fisheries and Life Science, Dalian Ocean University, Dalian 116023, China; 15941619221@163.com; 3 Industry Institute of Marine Ranching, Dalian Ocean University, Dalian 116023, China

**Keywords:** *Apostichopus japonicus*, artificial reef, bacterial community, environmental sources, tissue-specific selection

## Abstract

The sea cucumber *Apostichopus japonicus* is an important aquaculture species in artificial reef areas. Understanding the bacterial communities in its different tissues and their relationship with the environment is of great ecological significance for reef area management and healthy aquaculture. In this study, environmental samples, including seawater and sediment, as well as tissue samples of *A. japonicus* gut and respiratory tree, were collected from seven stations in the artificial reef area of Laizhou Bay, Bohai Sea. Total DNA was extracted from each sample, and the bacterial communities were characterized using PacBio full-length 16S rRNA amplicon sequencing technology. The results showed that bacterial diversity was highest in sediments, followed by the gut, whereas seawater and respiratory tree samples showed lower diversity. The gut bacterial community structure closely resembled that of the surrounding sediments, characterized by a substantial enrichment of taxa such as Cyanobacteriota and Planctomycetota. FAPROTAX analysis exhibited relatively high representation of putative functions related to complex organic matter degradation in gut samples. In contrast, the respiratory tree bacterial community structure was more similar to that of seawater, with Alphaproteobacteria as the dominant group. The respiratory tree samples showed relatively high predicted representation of methylotrophy and methanol oxidation, suggesting a putative functional association with one-carbon compound utilization. These results indicate that the bacterial community in *A. japonicus* tissues is shaped by a combination of environmental sources and tissue-specific selection, with the gut community being more closely associated with sediment and the respiratory tree community with seawater. These findings provide microecological insights into habitat optimization and sea cucumber stock enhancement management in artificial reef areas, specifically indicating that sediment- and seawater-derived microbial sources should be taken into account to support the tissue-specific bacterial communities of *A. japonicus*.

## 1. Introduction

In recent years, overfishing, eutrophication, climate change, physical disturbances and other factors have had a significant impact on China’s offshore fishery resources [1,2]. Laizhou Bay, located in the southern Bohai Sea, is a typical semi-enclosed bay that provides critical spawning and nursery habitats for numerous fishery species. However, under the influence of human and natural factors, the fishery resources and ecosystem structure of Laizhou Bay have undergone significant changes, with catches shifting towards lower trophic levels, smaller individuals, and lower-value species, accompanied by a decline in nearshore fishery productivity [3]. Artificial reefs are an effective way to protect and restore marine ecological functions [2]. They can improve habitat conditions and promote fishery resource conservation by providing shelter, feeding, and spawning habitats for marine organisms [4]. Reef structures can also modify local hydrodynamics and influence nutrient transport and material exchange across the sediment-water interface [5]. Thus, artificial reef construction is associated not only with fishery resource restoration but also with changes in benthic habitats and ecosystem processes in reef areas [6].

Sea cucumber (*Apostichopus japonicus*) is an important benthic species for stock enhancement in the marine ranching of northern China, and its resource enhancement is closely related to benthic habitat construction in reef-deployed areas [7]. As a typical benthic deposit-feeding sea cucumber, *A. japonicus* forages near the sediment-water interface and obtains nutrients by ingesting surface sediments, organic matter, bacteria, and other components [8]. Consequently, substrate type and sediment particle conditions are critical factors affecting its habitat suitability and stock enhancement [9]. It is noteworthy that the gut and respiratory tree of *A. japonicus* are two tissue compartments that are closely connected to sediment and seawater, respectively. The gut constantly receives material input from sediment, while the respiratory tree, which has the functions of respiration and water exchange, maintains long-term contact with the surrounding seawater [10].

Bacterial communities are crucial biological factors that influence both the external habitat conditions and the internal microecology of *A. japonicus*. Studies on the environmental sources of sea cucumber-related bacterial communities have primarily focused on the differences between water and sediment bacterial communities in aquaculture systems [11,12,13,14]. These studies showed that water and sediment bacterial communities differ in diversity, community composition, and predicted functions, suggesting that they may provide different external sources for sea cucumber-associated bacterial communities. Since *A. japonicus* is a deposit-feeding species, the relationship between sediment bacterial communities and gut bacterial communities has become a key research focus. Previous studies have shown that shared bacterial taxa and community correlations exist among gut, water, and sediment bacterial communities of *A. japonicus*, whereas different sample types still maintain distinct community compositions and functional profiles [15,16]. Gao et al. [15] compared the gut contents and surrounding sediments of *A. japonicus* using 16S rRNA gene pyrosequencing, and they found that the two shared some bacterial taxa, but the richness, diversity, and community structure of bacteria in the gut contents differed from those in sediment samples, indicating that the gut bacterial community of *A. japonicus* is associated with sediment but is not a direct replica of the sediment bacterial community. Similar results were also reported in a comparison between sea cucumber feces and habitat sediments, which shared many OTUs but still differed in community structure [17]. These findings indicated that sediment was one of the important external sources of gut and fecal bacterial communities in sea cucumbers, and that the composition of gut-associated bacterial communities was distinctly different from that of bacterial communities in sediment. This gut-sediment relationship is consistent with the sediment feeding and nutrient utilization of sea cucumbers. Pan et al. [18], through multi-omics and digestive physiological analyses, found that host digestive enzymes, gut structure, and symbiotic microorganisms in sea cucumbers are all associated with the utilization of sediment-derived nutrients, providing more direct biological evidence for understanding the relationship between deposit-feeding habits and gut-associated microorganisms. The environmental association with the bacterial community of sea cucumbers is not limited to the gut and sediment. Studies involving water, sediment, gut, and body surface samples showed that different sample types share bacterial taxa and community correlations while maintaining relatively specific community compositions [19]. Chung et al. [20] further found that host-associated samples, such as the body surface and gut of sea cucumbers, differed from surrounding environmental samples in community composition and predicted functions, indicating that both the external environment and host body site influence sea cucumber-associated bacterial communities.

Current research on bacterial communities associated with the sea cucumber *A. japonicus* has mainly focused on the gut, feces, body surface, and surrounding water and sediments [15,17,19,20]. In contrast, our understanding of bacterial communities related to the respiratory tree remains limited, especially regarding their relationship with the surrounding seawater. From the broader perspective of marine animal microbiome research, host-associated microbiomes can participate in host nutrient utilization, environmental adaptation, and ecosystem functioning [21]. Body surfaces, gills, and guts of marine animals are continuously exposed to the external environment, and their microbial community assembly is usually influenced by both environmental inputs and host tissue characteristics [22]. Studies on benthic animals such as oysters also show that different tissues can maintain relatively stable tissue-specific bacterial communities [23]. For *A. japonicus*, which is continuously exposed to sediment and seawater, whether the gut and respiratory tree form distinct associations with different environmental bacterial reservoirs and whether tissue-associated bacterial communities develop tissue-specific enrichment patterns based on environmental bacterial inputs remain to be further clarified.

In this study, seven sampling stations were selected in the artificial reef areas of Laizhou Bay, Bohai Sea. Environmental samples, including seawater and sediment, as well as tissue samples of *A. japonicus* gut and respiratory tree, were collected from each station. Using PacBio full-length 16S rRNA gene amplicon sequencing, we compared bacterial community composition, diversity, community structure, shared and unique taxa, differentially enriched taxa, and predicted functional features among the four sample types. This study aims to characterize the bacterial communities in the gut and respiratory tree of *A. japonicus*, as well as in seawater and sediment environments of the Laizhou Bay artificial reef areas, and further analyze the associations between bacterial communities in different tissues and environmental conditions. The results may provide direct microecological insights for sea cucumber stock enhancement management and habitat optimization in artificial reef areas.

## 2. Materials and Methods

### 2.1. Sample Collection

**Figure 1:**
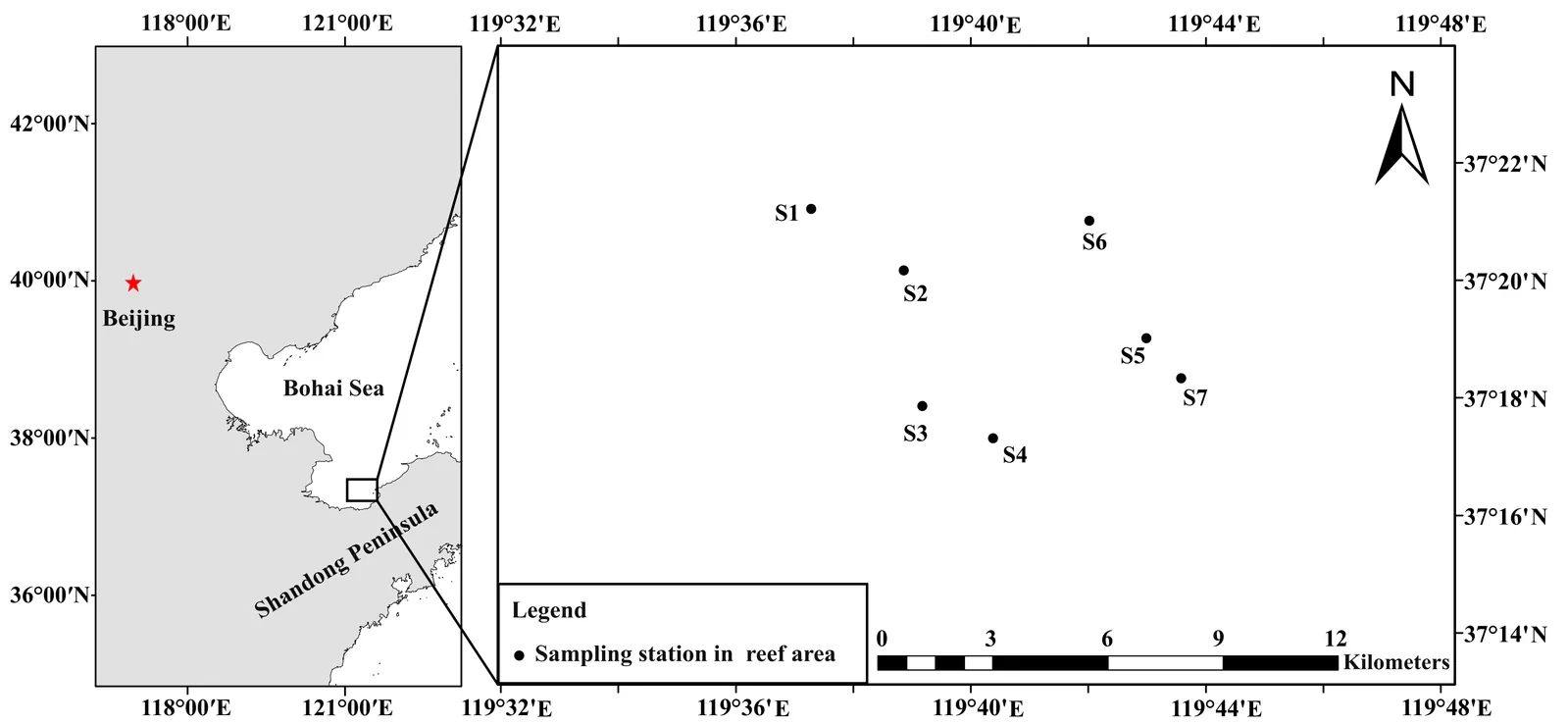
Map of the seven sampling stations in the artificial reef areas of Laizhou Bay, Bohai Sea.

As shown in Figure 1, the study area was located in the artificial reef areas of Laizhou Bay, Bohai Sea, China. In October 2025, seven sampling stations (S1–S7) were established in this area, and bottom seawater, surface sediment, and healthy adult *A. japonicus* were collected at each station. The sampled individuals were approximately 5 years old, with body lengths of 15–20 cm and wet body weights of 150–165 g. The geographic coordinates and water depth of stations S1–S7 are provided in Table S1. Based on sample source and tissue type, the samples were classified into four groups: seawater samples (SW), surface sediment samples (SED), gut samples (GUT), and respiratory tree samples (RT). Each station yielded four samples, comprising one SW sample, one SED sample, one GUT sample, and one RT sample, for a total of 28 samples for sequencing. For seawater samples (SW), three bottom seawater subsamples of 1 L each were collected at each station using a sterile water sampler. The subsamples were separately transferred into sterile polyethylene bottles and temporarily stored in an ice-cooled container. After transport to the laboratory, the three seawater subsamples from each station were separately filtered through 0.22 μm sterile polycarbonate membrane filters. The resulting filters were pooled for DNA extraction and treated as one composite SW sample for the station. For sediment samples (SED), approximately 1 kg of surface sediment was collected at each station using a grab sampler. The samples were placed in sterile sealed plastic bags and temporarily stored in an ice-cooled container. After transport to the laboratory, 2–3 g of surface sediment from each station was transferred into a 50 mL sterile centrifuge tube and stored at −80 °C as one SED sample for that station. Healthy adult *A. japonicus* individuals were randomly collected by divers near each station, with three individuals collected at each station. After collection, the individuals were immediately placed in clean containers filled with in situ seawater, temporarily stored in an ice-cooled container, transported alive to the laboratory, and dissected. Gut (GUT) and respiratory tree (RT) tissues were separately dissected from each individual under aseptic conditions. The tissue samples were gently rinsed with sterile water to remove loosely attached surface material. Tissues of the same type from the three individuals collected at the same station were pooled in equal amounts to generate one composite GUT sample and one composite RT sample for that station. The composite tissue samples were then aliquoted into 20 mL sterile centrifuge tubes, rapidly frozen in liquid nitrogen, and stored at −80 °C for subsequent DNA extraction and sequencing analysis.

### 2.2. Environmental Parameter Measurements

Water temperature, dissolved oxygen (DO), salinity, and pH were measured in situ at each sampling station using a YSI ProDSS multiparameter water quality meter (YSI, Yellow Springs, OH, USA). Water depth was measured using a conductivity–temperature–depth (CTD) profiler, and water transparency was determined using a Secchi disk and expressed as Secchi disk depth (SDD).

Seawater chemical parameters included chemical oxygen demand (COD), phosphate phosphorus (PO_4_-P), dissolved inorganic nitrogen (DIN), total nitrogen (TN), and total phosphorus (TP). COD was determined using the alkaline potassium permanganate method according to GB 17378.4-2007 [24]. Nutrient parameters were analyzed according to GB/T 12763.4-2007 [25]. Specifically, PO_4_-P was determined using the ascorbic acid reduction–phosphomolybdenum blue method; nitrite nitrogen (NO_2_-N), nitrate nitrogen (NO_3_-N), and ammonium nitrogen (NH_4_-N) were determined using the diazotization–azo method, zinc–cadmium reduction method, and hypobromite oxidation method, respectively. DIN was calculated as the sum of NO_3_-N, NO_2_-N, and NH_4_-N, whereas TN and TP were determined using the potassium persulfate oxidation method. For sediment samples, total organic carbon (TOC), TN, TP, and mean grain size were determined. TOC was measured using the potassium dichromate oxidation–reduction volumetric method, TP using the spectrophotometric method, and TN using the Kjeldahl titration method according to GB 17378.5-2007 [26]. Sediment grain size was determined according to GB/T 12763.8-2007 [27], and the mean grain size was used for subsequent analyses. The physicochemical parameters of seawater and sediment measured at stations S1–S7 are provided in Table S1.

### 2.3. DNA Extraction and PCR Amplification

The seawater filters were cut into small pieces, and total DNA was extracted from the seawater, sediment, gut and respiratory tree tissues using the TIANGEN Magnetic Soil and Stool Genomic DNA Kit (DP712; TIANGEN, Beijing, China). All extraction steps were performed according to the manufacturer’s instructions. The extracted DNA was quantified using the Qubit dsDNA HS Assay Kit (Thermo Fisher Scientific (Invitrogen), Eugene, OR, USA), and DNA integrity was assessed by agarose gel electrophoresis. The bacterial 16S rRNA gene was amplified using the primers 27F (5′-AGA GTTTGATCCTGGCTCAG-3′) and 1492R (5′-GGTTACCTTGTTACGACTT-3′) [28]. Each 25-μL PCR reaction contained 12.5 μL of 2× KAPA HiFi HotStart ReadyMix (Roche Sequencing Solutions, Cape Town, South Africa), 1 μL of each forward and reverse primer (10 μM), 10 ng of template DNA, and sterile water to a final volume of 25 μL. During DNA extraction and PCR amplification, negative controls were processed in parallel with the samples at a 1:1 ratio (one blank per sample) to monitor potential contamination. PCR was performed using the following conditions: initial denaturation at 95 °C for 5 min; 20–25 cycles of denaturation at 98 °C for 30 s, annealing at 57 °C for 30 s, and extension at 72 °C for 60 s, followed by a final extension at 72 °C for 5 min. The PCR products were purified using AMPure beads (Beckman Coulter Life Sciences, Brea, CA, USA), quantified using Qubit (Thermo Fisher Scientific (Invitrogen), Eugene, OR, USA), and verified by agarose gel electrophoresis. The purified amplicons from different samples were then pooled for Kinnex-based library preparation (Pacific Biosciences of California, Inc. (PacBio), Menlo Park, CA, USA). The resulting libraries were sequenced on the PacBio Revio platform (Pacific Biosciences of California, Inc. (PacBio), Menlo Park, CA, USA) using the Revio™ polymerase kit (Pacific Biosciences of California, Inc. (PacBio), Menlo Park, CA, USA). The sequencing data were used for subsequent bioinformatics analyses.

### 2.4. Data Processing and Visualization

Raw sequencing data generated on the PacBio Revio platform (Pacific Biosciences of California, Inc. (PacBio), Menlo Park, CA, USA) were processed using the SMRT Link (v10.0) pipeline to generate circular consensus sequencing (CCS) reads. The CCS reads were demultiplexed using lima module implemented in SMRT Link (v10.0) based on barcode sequences, and length filtering was performed using PRINSEQ version (v0.20.4), retaining sequences of 1000–1600 bp for subsequent analyses. After quality control, the sequences were processed using DADA2 (v1.14.0) [29] for denoising, chimera removal, and dereplication to obtain amplicon sequence variants (ASVs) and construct an ASV abundance table [30]. Representative ASV sequences were taxonomically annotated using the RDP Classifier (v2.12) [31] against the SILVA 16S rRNA gene database [32].

First, shared and unique ASVs among four sample types were analyzed using Venn diagrams, which were generated with the VennDiagram package (v1.6.20) in R (v3.6.0). Rarefaction curves were subsequently plotted using the vegan package (v2.5-6) in R (v3.6.0) based on changes in ASV number at different sequencing depths. Bacterial community composition was then summarized based on relative abundance matrices at the phylum, class, and genus levels. For the community composition plots, the ten most abundant taxa at each displayed taxonomic level were shown individually, while the remaining taxa were combined and presented as “Other”. At the phylum level, sample clustering and relative abundance patterns were jointly examined based on community compositional similarity and the relative abundance matrix. Sample clustering trees were constructed using the hclust function in the stats package in R (v3.6.0) and displayed together with the corresponding relative abundance bar plots. Relative abundance plots at the class and genus levels were generated using the ggplot2 package (v3.3.0) in R (v3.6.0). Next, alpha diversity indices, including Chao1, ACE, Shannon, Simpson, and Good’s coverage, were calculated using mothur (v1.43.0) and visualized using ggplot2. Pairwise differences in alpha-diversity indices among sample types were evaluated using *t*-tests, with Holm adjustment applied for multiple comparisons; adjusted *p* < 0.05 was considered statistically significant. To further evaluate differences in bacterial community structure. Pearson correlation analyses were performed separately for SW and SED samples to evaluate the relationships between alpha-diversity indices (ACE, Chao1, Shannon, and Simpson) and the corresponding environmental variables across the seven sampling stations. Pearson’s correlation coefficient (R) and the corresponding two-tailed *p* value were calculated, with *p* < 0.05 considered statistically significant. Bray–Curtis distance matrices were calculated from the ASV abundance matrix using the vegan package (v2.5-6) in R (v3.6.0), followed by principal coordinate analysis (PCoA) with the ordination visualized using ggplot2. Differences in bacterial community structure among sample types were tested using PERMANOVA based on Bray–Curtis distances, and the proportion of community variation explained by sample type was calculated using the vegan package (v2.5-6) in R (v3.6.0). Subsequently, differential taxa were identified using LEfSe (v1.1.0). LEfSe used the Kruskal–Wallis rank-sum test to screen taxa with significant differences among groups, followed by linear discriminant analysis (LDA) to evaluate their contribution to group separation with an LDA score threshold of 4.5. The results were presented as LDA barplots. Finally, potential ecological functions of the bacterial communities were predicted using FAPROTAX (v1.2.1), and functional abundance tables were generated for each sample. Functional abundance patterns were visualized as heatmaps using the gplots package (v3.0.1.1) in R (v3.6.0).

## 3. Results

### 3.1. Overview of Sequencing Data

**Figure 2:**
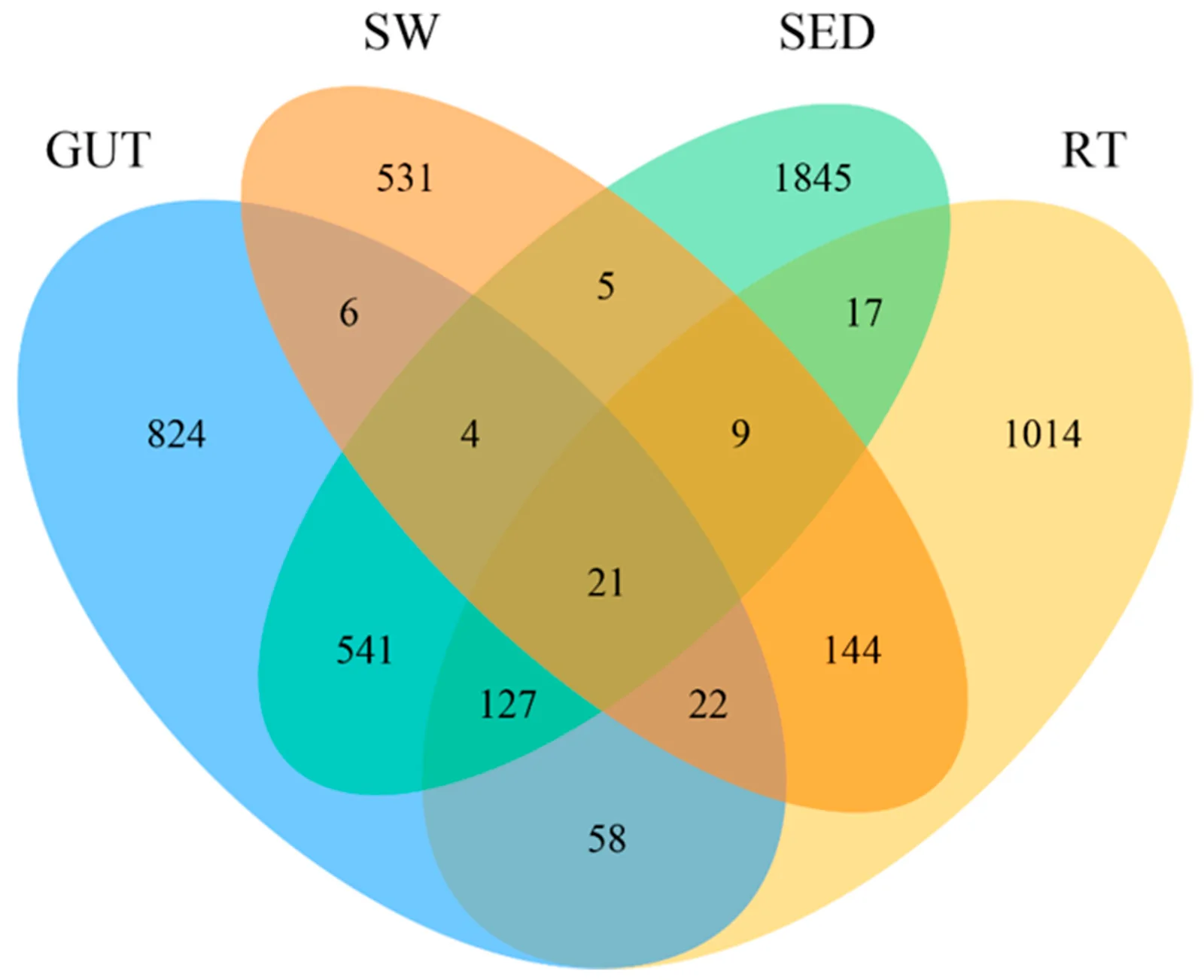
The Venn diagram showing the distribution of ASVs across the four sample types.

As shown in Table S2, the sequencing platform generated a total of 2,679,552 raw reads for the entire dataset. After quality filtering, 2,672,090 clean reads were retained, with an overall retention rate of 99.72%. The numbers of clean reads in GUT, RT, SED, and SW samples were 634,780, 664,263, 730,212, and 642,835, respectively. Across all samples, the number of clean reads ranged from 54,141 to 176,724, and Good’s coverage ranged from 96% to 100%. After clustering, a total of 5168 ASVs were obtained, with 1603, 1412, 2569, and 742 ASVs detected in GUT, RT, SED, and SW samples, respectively. Rarefaction curves showed that the number of ASVs in each sample gradually stabilized as the number of sampled sequences increased, further indicating that the sequencing depth was sufficient for subsequent bacterial community analysis (Figure S1). Venn diagram analysis at the ASV level showed a total of 21 shared ASVs across the four sample types. SED had the most unique ASVs (1845), followed by RT (1014) and GUT (824), while SW had the fewest unique ASVs (Figure 2). In pairwise comparisons, GUT and SED shared the largest number of ASVs, with 541 shared ASVs, followed by RT and SW with 144 shared ASVs. The remaining pairwise shared ASVs were 58 between GUT and RT, 17 between SED and RT, six between GUT and SW, and five between SW and SED.

### 3.2. Bacterial Community Composition and Differences Among the Four Sample Types

**Figure 3:**
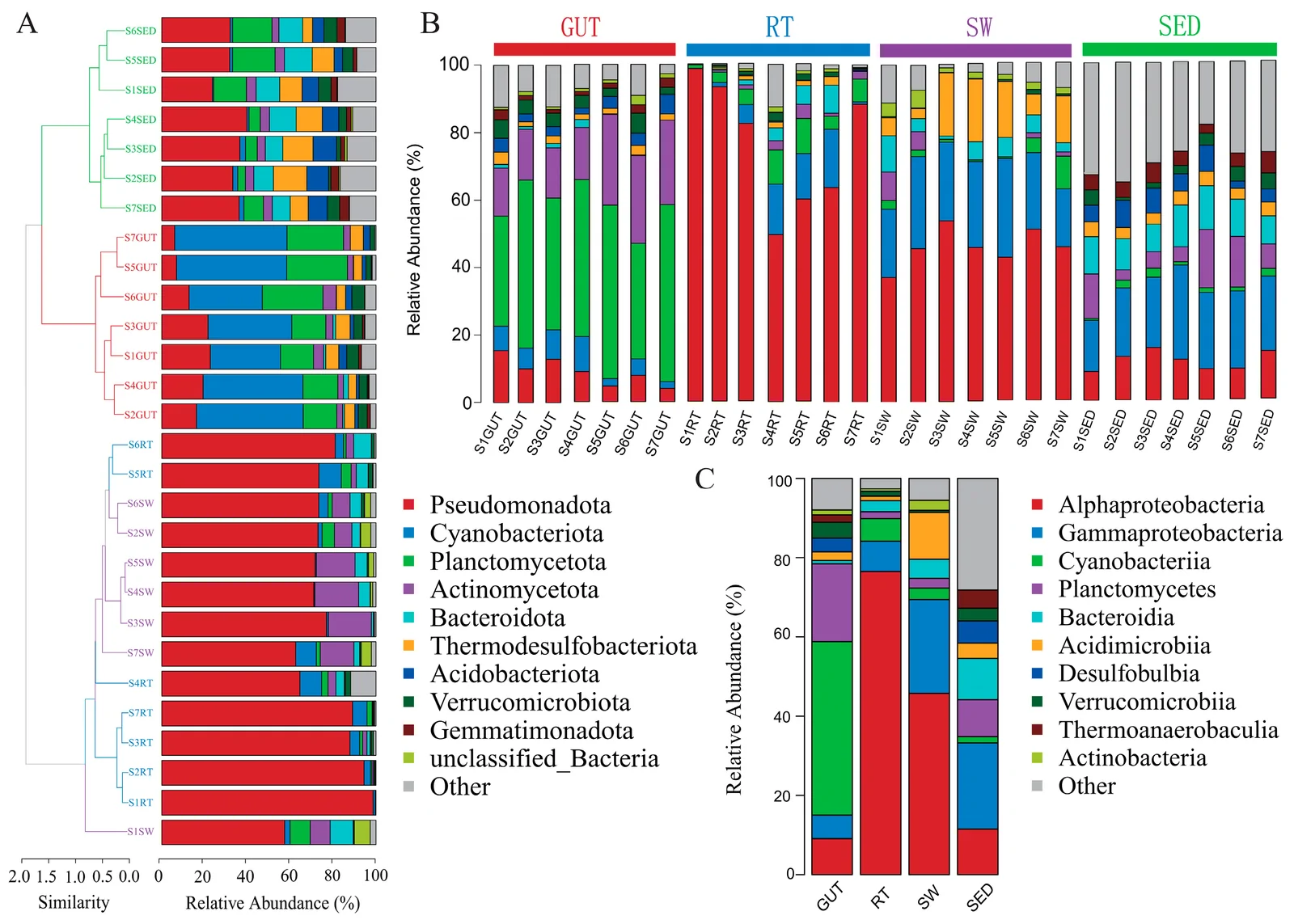
Relative abundance of bacterial communities at the phylum (**A**) and class (**B**,**C**) levels. Notes: (**A**) Bacterial community composition and sample clustering at the phylum level; (**B**) Bacterial community composition at the class level in all samples; (**C**) Bacterial community composition at the class level in four sample types. “Other” represents taxa outside the ten most abundant categories at the corresponding taxonomic level.

The results of phylum-level bacterial community composition analysis are shown in Figure 3A. Pseudomonadota accounted for the highest proportions in RT and SW samples, with relative abundances of 84.13% and 69.37%, respectively, whereas its proportions were lower in GUT and SED samples, at 15.07% and 33.26%, respectively. Planctomycetota showed higher relative abundances in GUT and SED samples, at 20.90% and 10.98%, respectively, but lower abundances in RT and SW samples, at only 1.89% and 2.73%, respectively. In addition, Cyanobacteriota was the most abundant phylum in GUT samples, accounting for 43.76%; Actinomycetota showed a relatively high abundance in SW samples, at 14.31%; and Thermodesulfobacteriota and Bacteroidota were relatively abundant in SED samples, at 10.81% and 10.47%, respectively. The sample clustering tree showed that the seven SED samples clustered within the same branch, and the seven GUT samples also clustered within the same branch; the SED and GUT samples then clustered together into a larger branch. RT and SW samples were located in another parallel branch, with some RT samples clustering close to SW samples.

At the class level, bacterial community composition further revealed both similarities and distinct features among the four sample types (Figure 3B,C). Alphaproteobacteria was the dominant class in RT and SW samples, with relative abundances of 76.52% and 45.74%, respectively, whereas its proportions were lower in GUT and SED samples, at only 9.15% and 11.57%, respectively. Planctomycetes showed higher relative abundances in GUT and SED samples, at 19.65% and 9.28%, respectively, but lower abundances in RT and SW samples, at only 1.72% and 2.44%, respectively. In addition, Cyanobacteria were the most abundant class in GUT samples, accounting for 43.74%. In RT samples, Gammaproteobacteria and Cyanobacteria accounted for 7.61% and 5.68%, respectively. In SW samples, Gammaproteobacteria and Acidimicrobiia showed relatively high abundances, at 23.63% and 11.77%, respectively. In SED samples, Gammaproteobacteria and Bacteroidia showed relatively high abundances, at 21.69% and 10.47%, respectively.

**Figure 4:**
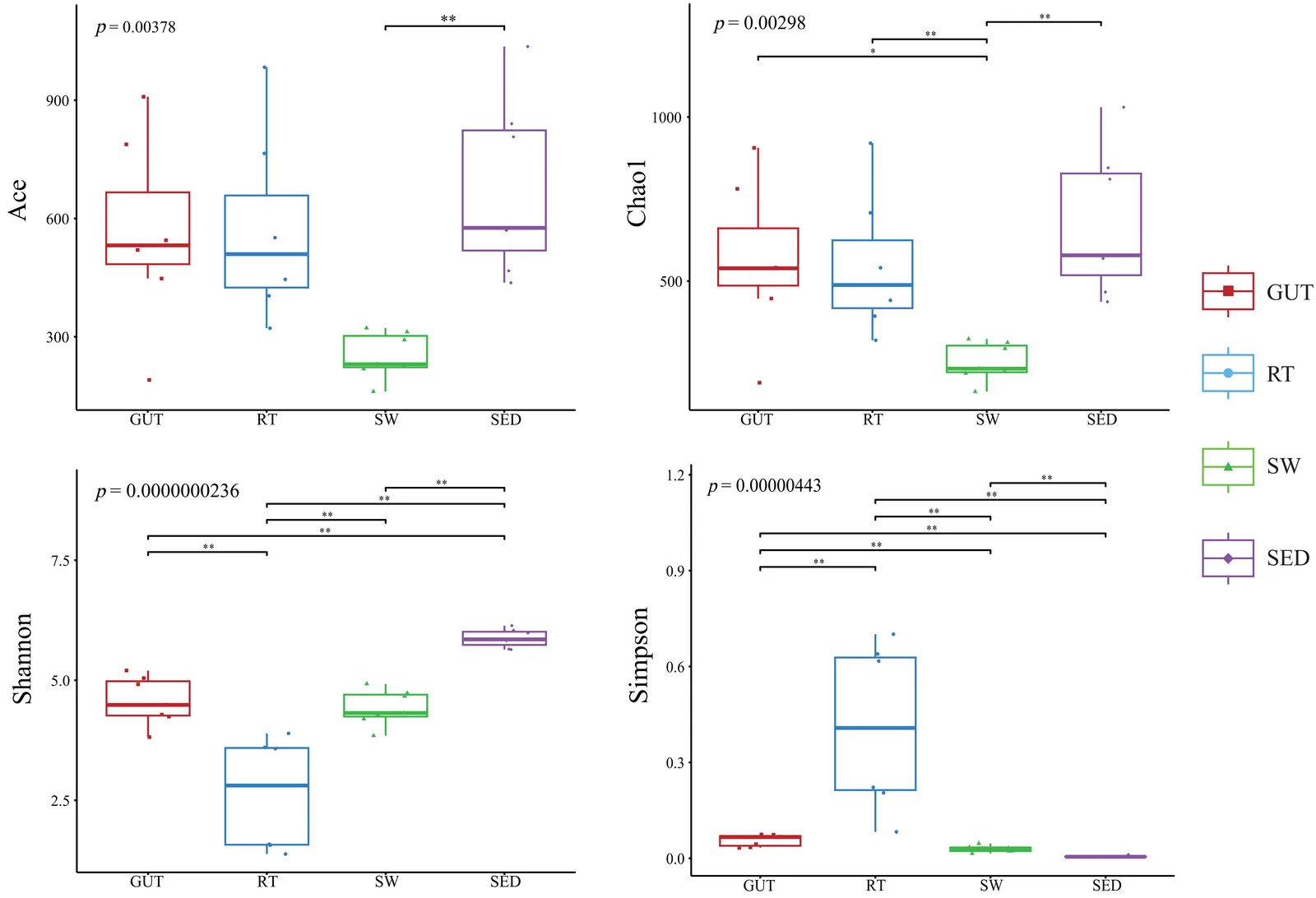
Alpha diversity indices of bacterial communities among the four sample types. Notes: * and ** indicate *p* < 0.05 and *p* < 0.01.

The results of alpha diversity analysis revealed differences in bacterial richness and diversity among the four sample types (Figure 4). Among the four sample types, the SED samples had the highest ACE, Chao1, and Shannon indices, indicating the highest bacterial richness and community diversity; SW samples showed lower ACE and Chao1 indices, whereas RT samples showed a lower Shannon index, indicating lower bacterial richness in SW samples and lower community diversity in RT samples, respectively. Between the two environmental sample types, the ACE, Chao1, and Shannon indices were significantly higher in SED samples than in SW samples (*p* < 0.05). Between the two tissue sample types, the Shannon index was significantly higher in GUT samples than in RT samples, whereas the Simpson index was significantly lower in GUT samples than in RT samples (*p* < 0.01). Comparisons among the four sample types showed that the ACE index was significantly higher in SED samples than in SW samples (*p* < 0.01); the Chao1 index was significantly higher in GUT, RT, and SED samples than in SW samples (*p* < 0.05); the Shannon index was significantly higher in SED samples than in GUT, RT, and SW samples, whereas it was significantly lower in RT samples than in GUT, SED, and SW samples (*p* < 0.01); and the Simpson index was significantly higher in RT samples than in GUT, SED, and SW samples (*p* < 0.01). Pearson correlation analysis showed that SDD was significantly positively correlated with the ACE, Chao1, and Shannon indices and significantly negatively correlated with the Simpson index (*p* < 0.05), whereas no significant correlations were detected between the measured sediment environmental variables and the four alpha-diversity indices (Table S3).

LEfSe analysis revealed differentially enriched bacterial taxa from the phylum to genus levels across the four sample types (Figure S2). For bacterial communities, using an LDA score threshold of 4.5, LEfSe identified 10, 5, 4, and 13 differentially enriched bacterial taxa in GUT, RT, SED, and SW samples, respectively. In GUT samples, the representative enriched taxa mainly included Cyanobacteriota, Planctomycetota, Cyanobiaceae, and Pirellulaceae. In RT samples, the representative enriched taxa mainly included Pseudomonadota, Alphaproteobacteria, Rhodobacterales, and Paracoccaceae. In SED samples, the representative enriched taxa mainly included Bacteroidota, Bacteroidia, Acidobacteriota, and Thermodesulfobacteriota. In SW samples, the representative enriched taxa mainly included Actinomycetota, Gammaproteobacteria, Pelagibacterales, and Pseudomonadales. At the genus level, the enriched taxa in GUT samples were norank_Cyanobiaceae and norank_Pirellulaceae; the enriched taxon in RT samples was norank_Paracoccaceae; and the enriched taxa in SW samples were norank_Pelagibacterales, norank_Pseudomonadales, and *Candidatus_Actinomarina*.

### 3.3. Associations Between Tissue-Associated and Environmental Bacterial Communities and Predicted Functional Profiles

**Figure 5:**
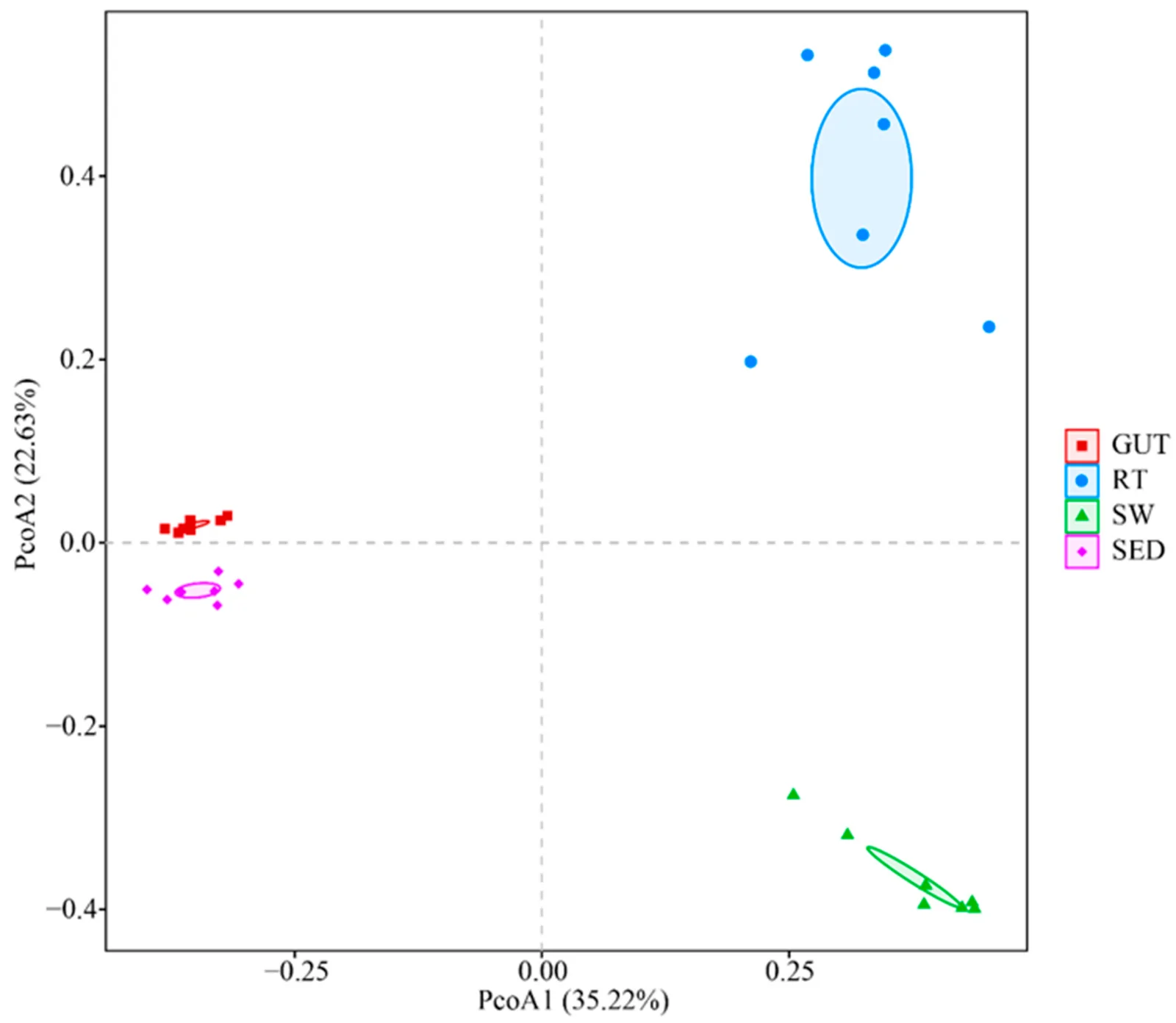
Principal coordinate analysis (PCoA) based on Bray–Curtis distances.

The results of PCoA based on Bray–Curtis distances revealed clear clustering patterns in bacterial community structure among the different sample types (Figure 5). PCoA1 and PCoA2 explained 35.22% and 22.63% of the community variation, respectively, with a cumulative explanation of 57.85%. GUT, RT, SW, and SED samples generally formed relatively independent clusters in the ordination space, with clear separation among the different sample types. Meanwhile, the bacterial community structures of GUT and SED samples were relatively close, and RT and SW samples also showed relatively high similarity, while samples within each sample type clustered closely.

The PERMANOVA results shown in Table 1 indicated that sample type had a significant effect on bacterial community structure (R^2^ = 0.766, *p* < 0.01). Pairwise comparisons showed significant differences in bacterial community structure between all pairs of sample types among GUT, RT, SW, and SED samples (*p* < 0.01). Among these comparisons, the R^2^ value was highest between GUT and RT (R^2^ = 0.781) and lowest between RT and SW (R^2^ = 0.587); the R^2^ value between GUT and SED was 0.673, which was lower than those for GUT versus RT and GUT versus SW. The PERMANOVA results were generally consistent with the grouping patterns of different sample types in the PCoA ordination.

**Figure 6:**
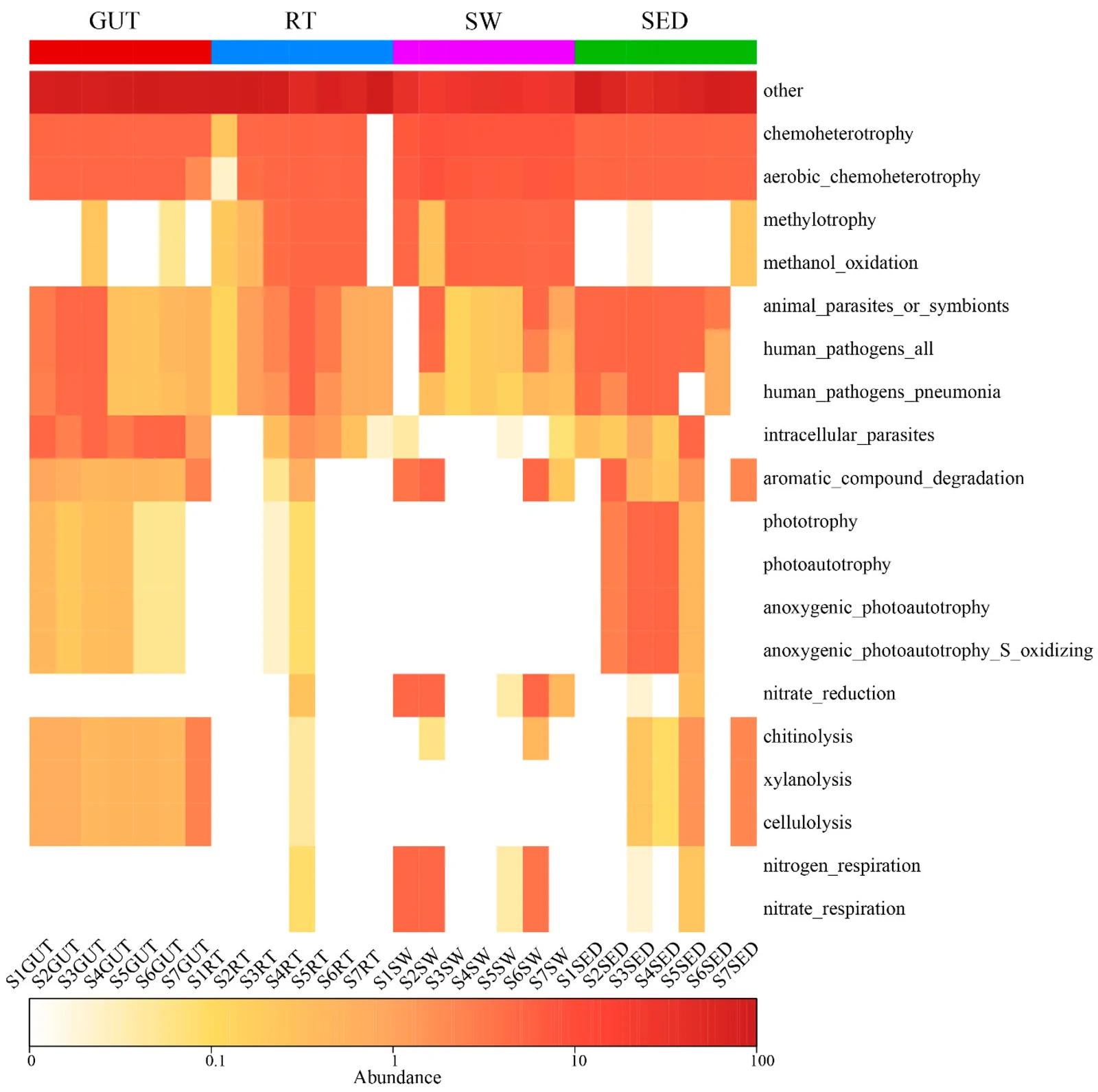
Predicted functional profiles of bacterial communities based on FAPROTAX.

The FAPROTAX-based functional abundance heatmap showed differences in the predicted functional profiles of bacterial communities among the four sample types, mainly involving functions related to carbon metabolism, nitrogen metabolism, sulfur cycling, and organic matter degradation (Figure 6). In SW samples, chemoheterotrophy and aerobic_chemoheterotrophy showed relatively high abundances, and nitrogen metabolism-related functions, including nitrate_reduction, nitrogen_respiration, and nitrate_respiration, were also prominent. In RT samples, methylotrophy and methanol_oxidation showed relatively high abundances; notably, these functions also showed high abundances in SW samples, indicating similar distribution patterns related to methylotrophy and methanol oxidation between RT and SW samples. In GUT samples, organic matter degradation-related functions, including chitinolysis, xylanolysis, and cellulolysis, showed relatively high abundances. In SED samples, functions related to phototrophy and sulfur oxidation, including phototrophy, photoautotrophy, anoxygenic_photoautotrophy, and anoxygenic_photoautotrophy_S_oxidizing, showed high abundances in some samples. Notably, chitinolysis, xylanolysis, and cellulolysis were also detected in some SED samples, indicating partial overlap with GUT samples in organic matter degradation-related functions.

## 4. Discussion

This study compared bacterial community composition, diversity, community structure, shared taxa, and predicted functional features among seawater, sediment, gut, and respiratory tree samples collected from the artificial reef areas of Laizhou Bay. The results showed clear differentiation in bacterial communities among the different sample types, with sediment and seawater forming distinct environmental bacterial community profiles. In terms of tissue-environment associations, the gut bacterial community of *A. japonicus* was more closely related to the sediment bacterial community, whereas the respiratory tree bacterial community showed greater similarity to the seawater bacterial community. Meanwhile, significant differences remained between the bacterial communities of the gut and respiratory tree and those of their corresponding environmental samples, indicating that tissue-associated bacterial communities within *A. japonicus* are not simple replicas of environmental bacterial communities but instead exhibit tissue-associated community differentiation and potential functional differences based on environmental sources. Based on these findings, the following discussion focuses on three aspects: bacterial community differences among sample types, associations between tissue-associated and environmental bacterial communities, and tissue-specific selective enrichment and predicted functional differentiation of bacterial communities in different tissues of *A. japonicus*.

### 4.1. Differences in Bacterial Community Characteristics Among Sample Types

In this study, the four sample types showed clear differences in bacterial richness and diversity, with SED generally exhibiting higher bacterial richness and diversity than SW. In addition, SDD was significantly associated with alpha diversity in SW, whereas no significant associations were detected for the measured sediment variables. Zhao et al. [14] compared bacterial communities in water and sediment from *A. japonicus* culture ponds and found higher bacterial diversity in sediment samples than in water samples. Zhou et al. [19] compared bacterial communities in water, sediment, and gut samples from an *A. japonicus* culture pond ecosystem and found that bacterial richness and diversity were highest in sediment, followed by gut and water samples. These results are consistent with the higher diversity in SED samples and lower richness in SW samples observed in the present study. The different environment–diversity patterns observed in SW and SED are also consistent with the contrasting habitat characteristics of the two environmental compartments: sediments contain abundant particulate matter, organic detritus, and microscale redox gradients that create heterogeneous bacterial niches [33,34,35], whereas seawater is more dynamic and its bacterial communities are more closely associated with dissolved and particulate organic matter transformation and nutrient cycling [36,37,38]. Beyond these environmental samples, GUT and RT also exhibited distinct diversity patterns, with GUT showing higher community diversity and RT showing greater dominance of specific taxa and lower community evenness. Wang et al. [16] compared bacterial communities in the intestine, surrounding water, and sediment of *A. japonicus* and found differences in bacterial richness, diversity, dominant taxa, and predicted functional pathways among the three sample types, which is consistent with the distinct bacterial community backgrounds among sample types observed in the present study. The gut of *A. japonicus* continuously receives inputs from sediments, organic detritus, and attached microorganisms [8], whereas the respiratory tree is involved in respiration and water exchange and is directly exposed to external seawater [10]. These differences in tissue function and environmental contact may be important factors shaping the distinct bacterial diversity patterns of GUT and RT samples.

Bacterial community composition and differentially enriched taxa further showed both shared and sample-specific characteristics among the four sample types. RT and SW shared dominance of Pseudomonadota and Alphaproteobacteria, whereas GUT and SED both showed relatively high abundances of Planctomycetota-related taxa; meanwhile, each sample type retained characteristic enriched taxa. Zhou et al. [19] reported that bacterial communities in water, sediment, and gut samples from *A. japonicus* culture ponds shared bacterial taxa and showed community correlations, while different sample types maintained specific dominant taxa and community compositions. This is consistent with the coexistence of shared taxa and clear community differentiation among the four sample types observed in the present study. Chung et al. [20] further compared bacterial communities in sea cucumber skin, gut, surrounding water, and sediment and found differences in community composition and predicted functions between host-associated and environmental samples, indicating that sea cucumber-associated bacterial communities can be influenced by both environmental filtering and host specificity. These findings also indicate that differences between tissue and environmental samples cannot be explained by a single environmental source alone, but should be interpreted in relation to the environmental exposure and physiological functions of different tissues. Based on these results, further comparisons of community structure and shared taxa among the gut, respiratory tree, seawater, and sediment can help clarify the corresponding relationships between bacterial communities in different tissues and bacteria derived from environmental sources.

### 4.2. Associations Between Tissue-Associated and Environmental Bacterial Communities in A. japonicus

This study further compared the associations between tissue-associated bacterial communities and environmental bacterial communities. GUT and SED samples showed relatively high similarity. Venn analysis showed that GUT and SED shared the largest number of ASVs among the pairwise comparisons. Their taxonomic composition also showed similarities, with both sample types containing relatively high abundances of Planctomycetota-related taxa. In addition, GUT and SED were positioned relatively close to each other in both the clustering analysis and PCoA ordination, while PERMANOVA showed lower explanatory power for the GUT–SED comparison than for GUT–RT and GUT–SW. Taken together, these results indicate that the gut bacterial community was more similar to the sediment bacterial community. Gao et al. [15] compared bacterial communities in the gut contents and surrounding sediments of *A. japonicus* and found that they shared some bacterial taxa and showed compositional links. Yamazaki et al. [17] also compared sea cucumber feces and habitat sediments and found that most fecal OTUs could be detected in sediment samples. These findings are consistent with the larger number of shared ASVs and the relatively close community structure between GUT and SED samples observed in the present study, further supporting the similarity between gut and sediment bacterial communities. This result is also consistent with the deposit-feeding habit of sea cucumbers, which mainly ingest sediments, organic detritus, and attached microorganisms, allowing sediment-derived particles and bacterial taxa to enter the gut during feeding [8,9]. From the perspective of sediment feeding and nutrient utilization, Pan et al. [18] showed that host digestive enzymes, gut structure, and symbiotic microorganisms in sea cucumbers are associated with the utilization of sediment-derived nutrients. Therefore, the high community similarity between GUT and SED samples in this study is consistent with the deposit-feeding habit and the continuous input of sediment-derived materials into the gut, indicating that sediment is an important environmental source of gut bacterial communities.

Corresponding to the GUT–SED relationship, RT and SW samples also showed relatively high similarity. Venn analysis showed that RT and SW shared the second-largest number of ASVs among the pairwise comparisons. Their taxonomic composition was also similar, with both sample types dominated by Pseudomonadota and Alphaproteobacteria. In addition, RT and SW were positioned relatively close to each other in the clustering analysis and PCoA ordination, while the RT–SW comparison showed the lowest explanatory power in the PERMANOVA analysis. Taken together, these results indicate that the respiratory tree bacterial community was more similar to the seawater bacterial community. The respiratory tree is an important organ for respiration and water exchange in sea cucumbers and is in long-term direct contact with external seawater [10], providing a basis for seawater-derived bacterial inputs to influence its bacterial community composition. Similarly, Sehnal et al. [39] noted in a review of microbiome composition and function in aquatic vertebrates that fish gills, as respiratory mucosal interfaces directly exposed to the aquatic environment, are continuously influenced by microbial inputs from surrounding water. Pratte et al. [40], based on 16S rRNA gene sequencing of reef fish gill microbiomes, found that 75–85% of fish-associated OTUs were also detected in environmental samples, including seawater, and suggested that fish gill microbiomes may be influenced by surrounding environmental microbial reservoirs. These findings are generally consistent with the larger number of shared ASVs, similar dominant taxa, and shorter community distance between RT and SW samples observed in the present study. The relatively high similarity between RT and SW samples is consistent with the tissue characteristics of the respiratory tree, which performs water exchange and is directly exposed to external seawater, suggesting that seawater may be an important environmental source of respiratory tree bacterial communities. Together with the similarity between GUT and SED samples, these results indicate that tissue-associated bacterial communities are related to the environments with which the tissues are in direct contact and are also influenced by tissue functional characteristics. Based on these associations, further integration of differentially enriched taxa and predicted functional results is needed to analyze the enrichment characteristics and functional differentiation of environmental bacteria after entering different tissues.

### 4.3. Tissue-Specific Enrichment and Predicted Functional Differentiation of Bacterial Communities in A. japonicus

Although GUT and SED samples showed relatively high similarity in community structure, the present study further showed that the GUT bacterial community was not a simple extension of the SED bacterial community, but developed clear gut-associated characteristics in terms of differentially enriched taxa and predicted functional profiles. Taxonomic composition showed that both GUT and SED samples contained relatively high abundances of Planctomycetota-related taxa, indicating that sediment-derived bacterial input contributes to the formation of the gut bacterial community. Further LEfSe analysis showed that GUT samples were enriched in Planctomycetota/Pirellulaceae and Cyanobacteriota/Cyanobiaceae from the phylum to family levels, suggesting that GUT maintained compositional associations with SED while forming differentially enriched taxa distinct from those in SED. Meanwhile, FAPROTAX-based functional prediction showed relatively high representation of chitinolysis, xylanolysis, and cellulolysis in GUT samples, suggesting a putative functional association with complex organic matter degradation. These results indicate that the formation of the GUT bacterial community is related not only to sediment-derived inputs under deposit-feeding conditions, but also to further selection by the gut nutritional environment and local microenvironment. Yamazaki et al. [17] compared microbial communities in sea cucumber feces and habitat sediments using 16S rRNA gene sequencing and suggested that the sea cucumber gut can selectively enrich sediment-associated microorganisms, indicating that the gut bacterial community is not a passive copy of the external sediment bacterial community. Pan et al. [18] further showed, from a genomic perspective, that sea cucumbers and their symbiotic microbiomes are adapted to the utilization of seabed sediment resources. These findings are consistent with the present result that GUT and SED samples were similar, while GUT samples had characteristic enriched taxa and predicted functional features. Planctomycetes have been reported as one of the dominant bacterial groups in the gut of *A. japonicus* [41]. These taxa have strong potential for polysaccharide degradation, and related representative taxa possess complex genomic structures and diverse metabolic capacities [42,43]. In addition, Pirellulaceae-related taxa have been reported to show higher relative abundance in the gut of faster-growing individuals and to be associated with bacteria involved in algal polysaccharide degradation [44]. Combined with the relatively high predicted representation of chitinolysis, xylanolysis, and cellulolysis in GUT samples, the enrichment of Planctomycetota and Pirellulaceae suggests a potential association between these taxa and complex organic matter degradation in the gut. In addition, the enrichment of Cyanobacteriota and the corresponding family-level taxon Cyanobiaceae further supports the contribution of deposit-feeding-derived inputs. Marine cyanobacteria are important contributors to marine primary production and can provide photosynthetic components for the formation of marine particulate organic matter [45]. During deposit feeding, the gut continuously contacts biogenic particles such as sediments, microalgae, and organic detritus [18], and Cyanobacteria-related taxa can therefore become part of the digestive tract-associated microbial community. Deng et al. [41] also reported that Cyanobacteria were components of the gut bacterial community of *A. japonicus* and showed seasonal variation in relative abundance, which is consistent with the present study. Therefore, the enrichment of Cyanobiaceae-related taxa mainly reflects the input background of sediments and particulate organic matter during deposit feeding. Collectively, these results indicate that the gut bacterial community is influenced by sediment-derived bacterial inputs and further exhibits enrichment of bacterial taxa and predicted functions related to complex organic matter degradation, reflecting tissue-associated selective enrichment of GUT distinct from SED.

RT and SW samples also showed relatively high similarity in community structure; however, the RT bacterial community was not a simple extension of the SW bacterial community, but developed clear respiratory tree-associated characteristics in terms of differentially enriched taxa and predicted functional profiles. Taxonomic composition showed that both RT and SW samples were dominated by Pseudomonadota and Alphaproteobacteria, indicating that seawater-derived bacterial input contributes to the formation of the RT bacterial community. Further LEfSe analysis showed that the differentially enriched taxa in RT were mainly concentrated in the Pseudomonadota-Alphaproteobacteria-Rhodobacterales-Paracoccaceae taxonomic branch, indicating that RT maintains compositional associations with SW while showing further enrichment within this lineage rather than simply inheriting the dominant seawater taxa. Pratte et al. [40] found that reef fish gills supported a distinct microbiome compared with environmental samples and identified gill-associated differentially abundant OTUs, which is consistent with the present finding that RT formed a characteristic enriched taxonomic branch against the background of SW-dominated taxa. Meanwhile, FAPROTAX-based functional prediction showed relatively high representation of methylotrophy and methanol_oxidation in RT samples, suggesting a potential association with one-carbon compound utilization. The respiratory tree is an important organ for respiration and water exchange in sea cucumbers and is in long-term direct contact with external seawater [10], providing a basis for the community similarity between RT and SW and for seawater-derived bacterial inputs. Within the enriched taxonomic branch in RT, Pseudomonadota and Alphaproteobacteria are located at higher taxonomic levels. Their enrichment is consistent with the shared dominant taxa between RT and SW and with the recognition of Alphaproteobacteria as an important component of marine bacterioplankton [46], indicating a clear seawater bacterial reservoir background for this enriched lineage in RT. At the order level, Rhodobacterales, an important marine-associated group within Alphaproteobacteria, are widely distributed in seawater, algal-associated environments, and particle-attached habitats and participate in marine organic matter transformation and carbon and sulfur cycling [47]. Their enrichment further reflects the link between the water-exchange interface of the respiratory tree and seawater-associated bacterial taxa. At the family level, Paracoccaceae-related taxa showed a clear association with the predicted potential for one-carbon compound utilization in RT. Huang et al. [48] classified Paracoccaceae and analyzed their genomic features using phylogenomic approaches, showing that Paracoccaceae-related taxa can occur in coastal marine environments and possess phylogenetic and metabolic diversity. Dziewit et al. [49] analyzed the methylotrophic characteristics of *Paracoccus aminophilus* JCM 7686 through whole-genome sequencing and comparative genomic analysis and showed that its genome contains genes related to the oxidation and assimilation of one-carbon compounds, such as methanol and methylamine, and that this bacterium can use one-carbon compounds as carbon and energy sources. Combined with the relative enrichment of methylotrophy and methanol_oxidation in RT samples in the present study, the enrichment of Paracoccaceae-related taxa is consistent with the predicted functional tendency toward one-carbon compound utilization in RT, suggesting that this group may serve as a potential indicator of one-carbon metabolic potential in RT. Therefore, the successive enrichment of the Pseudomonadota-Alphaproteobacteria-Rhodobacterales-Paracoccaceae taxonomic branch reflects the input background of the seawater bacterial reservoir to the RT bacterial community and is consistent with predicted functional patterns related to organic matter transformation and one-carbon compound utilization at the respiratory tree interface. These results indicate that the respiratory tree bacterial community is influenced by seawater-derived bacterial inputs and is further characterized by taxonomic enrichment associated with the respiratory tissue interface and predicted functional features related to one-carbon metabolic potential, reflecting tissue-associated selective enrichment of RT distinct from SW. Together with the enrichment patterns of the gut bacterial community, these results suggest that different tissues of *A. japonicus* can develop distinct taxonomic enrichment patterns and divergent predicted functional profiles under the influence of environmental bacterial sources, reflecting the combined effects of tissue structure, environmental exposure, and local microenvironments on bacterial community assembly.

## 5. Conclusions

This study analyzed the relationships among bacterial communities in the gut and respiratory tree of *A. japonicus* and those in surrounding seawater and sediment in the artificial reef areas of Laizhou Bay. The gut bacterial community showed a closer association with the surrounding sediment and was characterized by the enrichment of taxa such as Cyanobacteriota and Planctomycetota, whereas the respiratory tree bacterial community was more closely associated with seawater and was dominated by Alphaproteobacteria. FAPROTAX-based predictions further suggested potential functional differences between the two tissue-associated bacterial communities, particularly in relation to complex organic matter degradation in GUT and one-carbon compound utilization in RT. These results indicate that the bacterial community in *A. japonicus* tissues is jointly shaped by both environmental sources and tissue-specific selection. Future studies could further integrate quantitative analysis of functional genes, metagenomics, and metatranscriptomics to verify the functional processes and ecological significance of bacterial communities in different tissues. From an applied perspective, the tissue–environment associations and characteristic bacterial taxa identified here may provide useful microbial information for environmental monitoring in artificial reef areas, sea cucumber health assessment, and the optimization of stock enhancement and aquaculture management.

## Figures and Tables

**Table 1:** PERMANOVA analysis of bacterial community structure among sample types.

Groups	Sample Size	Group Size	F. Model	R^2^	*p* Value
GUT vs. RT	14	2	42.76	0.78	0.001
GUT vs. SW	14	2	27.53	0.70	0.001
GUT vs. SED	14	2	24.71	0.67	0.002
RT vs. SW	14	2	17.07	0.59	0.001
RT vs. SED	14	2	33.66	0.74	0.003
SW vs. SED	14	2	22.20	0.65	0.001
Between	16	4	26.21	0.77	0.001

## Data Availability

The original contributions presented in the study are included in the article/Supplementary Material, further inquiries can be directed to the corresponding authors.

## Supplementary Materials

The following supporting information can be downloaded at: https://www.mdpi.com/article/10.3390/microorganisms14092109/s1, Figure S1: Rarefaction curves of bacterial communities based on ASV numbers at different sequencing depths; Figure S2: Differentially enriched bacterial taxa identified by LEfSe analysis among the four sample types; Table S1: Geographic Coordinates and Environmental Factors of the Artificial Reef Area in Laizhou Bay, Bohai Sea; Table S2: Sequencing statistics, amplicon sequence variant (ASV) counts, and Good’s coverage for Apostichopus japonicus gut (GUT) and respiratory tree (RT) samples and surrounding sediment (SED) and seawater (SW) samples; Table S3: Pearson correlations between bacterial alpha diversity indices (ACE, Chao1, Shannon, and Simpson) and corresponding environmental variables in seawater (SW) and sediment (SED) samples.
