# Supplementary material for: Environmental Sources and Tissue-Specific Selection Jointly Shape the Bacterial Community of the Sea Cucumber (Apostichopus japonicus) in Artificial Reef Areas of Laizhou Bay, Bohai Sea
Source: Microorganisms. 2026 Sep 20;14(9):2109. doi: 10.3390/microorganisms14092109 (PMC13609560; doi:10.3390/microorganisms14092109)
Supplement: Supplementary file 1 [file microorganisms-14-02109-s001.zip › Table S1.pdf]

Table S1. Geographic Coordinates and Environmental Factors of the Artificial Reef Area in Laizhou Bay, Bohai Sea

Sampling station	Longitude (°E)	Latitude (°N)	Water											Sediment			
			T(°C)	DO(mg/L)	Sal	pH	SDD(m)	Depth(m)	COD (mg/L)	PO_4-P (mg/L)	DIN (mg/L)	TN (mg/L)	TP (mg/L)	TOC (%)	TP (mg/g)	TN (mg/g)	GS (μm)
S1	119°37.280	37°21.210	14.3	9.79	22.2	8.2	2.1	11.2	1.46	0.0061	0.181	0.96	0.035	0.809	0.492	0.526	71.5
S2	119°38.857	37°20.162	14.3	9.77	22.06	8.2	2	10.5	1.5	0.0068	0.252	1.02	0.038	1.047	0.479	0.616	50.0
S3	119°39.172	37°17.854	13.8	9.86	21.47	8.18	1.1	9.8	1.54	0.0089	0.178	1.12	0.043	1.021	0.450	0.613	46.8
S4	119°40.380	37°17.309	13.7	9.28	22.5	8.14	0.9	8.9	1.34	0.0137	0.221	1.28	0.046	0.802	0.471	0.628	67.2
S5	119°42.988	37°19.011	13.3	10.47	22.05	8.1	1.6	9.3	1.31	0.0064	0.239	1.08	0.037	0.764	0.457	0.527	64.3
S6	119°42.020	37°21.011	13.7	10.86	21.77	8.21	1.5	9.5	1.42	0.005	0.197	1.31	0.044	0.887	0.459	0.552	65.3
S7	119°43.578	37°18.327	13	9.46	22.34	8.13	1.23	8	1.51	0.0075	0.225	0.92	0.049	0.789	0.465	0.536	61.9

Notes: T, water temperature; DO, dissolved oxygen; Sal, salinity; SDD, Secchi disk depth; Depth, water depth; COD, chemical oxygen demand; PO_4-P, phosphate phosphorus; DIN, dissolved inorganic nitrogen; TN, total nitrogen; TP, total phosphorus; TOC, total organic carbon; GS, mean grain size.
