# Supplementary material for: Environmental Sources and Tissue-Specific Selection Jointly Shape the Bacterial Community of the Sea Cucumber (Apostichopus japonicus) in Artificial Reef Areas of Laizhou Bay, Bohai Sea
Source: Microorganisms. 2026 Sep 20;14(9):2109. doi: 10.3390/microorganisms14092109 (PMC13609560; doi:10.3390/microorganisms14092109)
Supplement: Supplementary file 1 [file microorganisms-14-02109-s001.zip › Table S2.pdf]

Table S2. Sequencing statistics, amplicon sequence variant (ASV) counts, and Good's coverage for Apostichopus japonicus gut (GUT) and respiratory tree (RT) samples and surrounding sediment (SED) and seawater (SW) samples.

Group	Sample	Raw reads	Clean reads	ASVs	Good's coverage
GUT	S1GUT	77706	77508	491	0.99
	S2GUT	79000	78758	421	0.99
	S3GUT	65718	65519	789	0.98
	S4GUT	75870	75710	502	0.99
	S5GUT	135485	135107	467	0.99
	S6GUT	77199	77020	714	0.98
	S7GUT	125488	125158	190	1.00
RT	S1RT	69339	69147	403	0.99
	S2RT	75380	75220	368	0.99
	S3RT	81507	81323	745	0.96
	S4RT	91648	91299	434	0.99
	S5RT	126071	125742	603	0.98
	S6RT	54319	54141	479	0.99
	S7RT	167822	167391	304	0.99
SED	S1SED	81157	80929	771	0.99
	S2SED	76530	76322	437	1.00
	S3SED	95984	95741	954	0.98
	S4SED	64713	64584	792	0.99
	S5SED	163091	162636	566	1.00
	S6SED	177300	176724	562	1.00
	S7SED	73459	73276	466	1.00
SW	S1SW	79244	79013	318	1.00
	S2SW	73270	73080	284	1.00
	S3SW	78619	78357	158	1.00

S4SW	133272	132842	216	1.00
S5SW	68772	68599	225	1.00
S6SW	142216	141760	309	1.00
S7SW	69373	69184	223	1.00
