# Supplementary material for: Environmental Sources and Tissue-Specific Selection Jointly Shape the Bacterial Community of the Sea Cucumber (Apostichopus japonicus) in Artificial Reef Areas of Laizhou Bay, Bohai Sea
Source: Microorganisms. 2026 Sep 20;14(9):2109. doi: 10.3390/microorganisms14092109 (PMC13609560; doi:10.3390/microorganisms14092109)
Supplement: Supplementary file 1 [file microorganisms-14-02109-s001.zip › Table S3.pdf]

Table S3. Pearson correlations between bacterial alpha diversity indices (ACE, Chao1, Shannon, and Simpson) and corresponding environmental variables in seawater (SW) and sediment (SED) samples.

Sea water environmental variable	Alpha diversity indices							
	ACE		Chao1		Shannon		Simpson	
	R	P	R	P	R	P	R	P
T	0.495	0.258	0.506	0.247	0.484	0.271	-0.460	0.299
DO	0.350	0.441	0.357	0.432	0.283	0.539	-0.231	0.618
Sal	0.198	0.671	0.189	0.684	0.249	0.591	-0.336	0.461
pH	0.560	0.191	0.560	0.191	0.524	0.227	-0.430	0.336
SDD	0.778	**0.039***	0.788	**0.035***	0.808	**0.028***	-0.822	**0.023***
Depth	0.529	0.223	0.543	0.208	0.535	0.215	-0.523	0.229
COD	-0.068	0.885	-0.073	0.877	-0.035	0.940	0.102	0.827
PO_4-P	-0.580	0.172	-0.584	0.169	-0.584	0.168	0.548	0.203
DIN	0.039	0.935	0.042	0.929	0.034	0.943	-0.104	0.824
TN	-0.059	0.899	-0.060	0.898	-0.166	0.722	0.218	0.638
TP	-0.454	0.306	-0.473	0.284	-0.475	0.281	0.514	0.238
Sediment environmental variable	Alpha diversity indices							
	ACE		Chao1		Shannon		Simpson	
	R	*p*	R	*p*	R	*p*	R	*p*
TOC	0.121	0.797	0.110	0.815	-0.250	0.588	0.587	0.166
TP	-0.146	0.754	-0.136	0.771	0.097	0.837	-0.517	0.234
TN	0.353	0.437	0.351	0.440	0.220	0.635	0.278	0.546
GS	-0.088	0.852	-0.074	0.874	0.372	0.411	-0.736	0.059

Notes: * indicate $P < 0.05$.
